# First Report of Swim Bladder-Associated Microbiota in Rainbow Trout (*Oncorhynchus mykiss*)

**DOI:** 10.1264/jsme2.ME17071

**Published:** 2017-10-14

**Authors:** Alejandro Villasante, Carolina Ramirez, Natalia Catalán, Jaime Romero

**Affiliations:** 1 Laboratorio de Biotecnología, Instituto de Nutrición y Tecnología de los Alimentos (INTA), Universidad de Chile Avda El Líbano 5524, Santiago Chile; 2 Laboratorio de Biotecnología, Unidad de Alimentos, Instituto de Nutrición y Tecnología de los Alimentos (INTA), Universidad de Chile Avda El Líbano 5524, Santiago Chile

**Keywords:** swim bladder, microbiota, rainbow trout, salmonids, symbiosis

## Abstract

The aim of the present study was to identify major bacteria associated with the swim bladder in the rainbow trout, *Oncorhynchus mykiss*. We extracted DNA from the swim bladder and gut contents in order to perform a temporal temperature gradient gel electrophoresis (TTGE) analysis of 16S rRNA amplicons for bacterial identification to further compare both profiles. *Arthrobacter* and *Cellulosimicrobium* were the major genera observed in the swim bladder in fish, but were not present in fish gut contents; *Mycoplasma* were instead observed in these samples. Further research to investigate the possible symbiotic roles of the swim bladder-associated microbiota in salmonids is needed.

In the last few decades, most research on fish microbiota has been conducted on the gut-associated microbiota in an attempt to elucidate its role in regulating physiology and promoting health in fish (9, 17). However, microbiota associated with anatomic compartments open to the environment other than the gastro-enteric system, including the swim bladder mucosa of physostomes, has received less attention. Few studies have focused on using the mucosa of the swim bladder as a model to investigate mucosal candidiasis in zebrafish larvae (7, 8).

The swim bladder is described as a homolog structure of the tetrapod lung, the primary function of which is to counteract the force of gravity by modulating whole body density, thereby providing an energetic and cost-efficient buoyancy-controlling system in fish (23, 26). Based on studies conducted in the zebrafish model, the swim bladder and lungs have been shown to share an embryonic origin, a single or paired pouch of the cranial part of the embryonic digestive tube (24, 27). From an evolutionary perspective, the tetrapod lung and teleost swim bladder have been proposed to diverge from a primitive fish lung, similar to that found in *Polypterus* (14). In teleost species, a direct connection exists between the swim bladder and esophagus via the pneumatic duct, which appears during the late embryonic stage (24). In physostomes, the pneumatic duct persists throughout the entire life cycle, while physoclists lose this connection after the first inflation of the swim bladder, and, thus, these species have a closed swim bladder during the juvenile and adult stages (25). In physostomes, including some of economic importance, such as salmonids and carp, the swim bladder is mainly filled with gas by “gulping” air from the environment. Some species may develop abnormal swim bladder distention due to the action of pathogens (*i.e.*, bacteria, fungi, and/or parasites) (18). The pneumatic duct appears to constitute the major route of access for these pathogens and, hypothetically, to commensal microbes that inhabit the swim bladder mucosa. Hence, we hypothesize that the swim bladder mucosa of physostomes may harbor ecological niches for commensal bacteria in healthy fish, and, thus, constitute a similar non-sterile structure to that reported in the human lung microbiome (3, 20).

To the best of our knowledge, the resident bacterial community of the swim bladder mucosa in physostomes, such as the rainbow trout, has not yet been reported. This topic requires further study in order to obtain a better understanding of the role of the swim bladder microbiota in regulating aspects of the immune system and physiology of physostomes, particularly in aquaculture species. Therefore, the main goal of the present study is to identify bacteria associated with the swim bladder, and compare them with distal gut bacteria obtained from the same host.

Ten fish (*Oncorhynchus mykiss*) were kept in a 100-L fiberglass tank supplied with well-aerated fresh water at a constant temperature (15±0.5°C, 8 L min^−1^). Fish were exposed to a constant photoperiod (14 h light/10 h dark) and fed a commercial diet three times every d for eight weeks. Five fish (50±5 g) were sampled to collect the swim bladder and intestinal contents. However, only four fish had intestinal contents at the time of sampling. Fish were euthanized as described by Navarrete *et al.* (2012) (13) and the recommendations of the Guide for the Care and Use of Laboratory Animals of the National Institutes of Health, and the Committee on the Ethics of Animal Experiments of Universidad de Chile. The swim bladder was aseptically dissected and washed in 15 mL of PBS, following by gentle vortexing at room temperature for 5 min. Washing was repeated twice. The cloudy liquid was transferred to 1.5-mL tubes and centrifuged at 13,000×*g* for 1 min. The resulting pellet was stored at −80°C until nucleic acid extraction. The intestinal contents were collected and flash frozen at −80°C until further analyses. Nucleic acid extraction was performed using swim bladder-derived pellets and intestinal contents with MoBio kit Power soil and MoBio kit Fecal, respectively (MoBio Laboratories, Carlsbad, CA, USA) according to the manufacturer’s instructions, with an initial enzymatic treatment using lysozyme and proteinase as described by Navarrete *et al.* (2010) (12). In order to obtain fingerprints of the bacterial communities present in the swim bladder and intestinal contents, the 16S rRNA gene was amplified by PCR and analyzed using temporal temperature gradient gel electrophoresis (TTGE), as described by Navarrete *et al.* (2012) (12). Dominant bands from TTGE gels were excised, eluted, and reamplified (12). Amplicons were sequenced by the Macrogen USA sequencing service (Rockville, MD, USA). 16S rRNA gene sequences were compared with those available in the Ribosomal Database Project II (http://rdp.cme.msu.edu/seqmatch/seqmatch_intro.jsp) in order to identify their closest relatives (Table 1). The sequences from TTGE bands obtained in this study have been deposited in the GenBank database under accession numbers MF787227 to MF787238.

We observed low bacterial diversity based on TTGE profiles in the swim bladder resident microbiota (Fig. 1). Two high-density bands most likely corresponding to *Arthrobacter globiformis* and *Cellulosimicrobium* sp. were detected in all analyzed fish (*n*=5). On the other hand, we identified higher diversity (more bands in the TTGE profile) in intestinal contents than in the swim bladder resident microbiota profile in all individuals (*n*=4) (Fig. 2). Several bacterial genera such as *Eubacterium*, *Pseudomonas*, *Aliivibrio*, and *Vibrio*, were detected in weak bands (d to j in Fig. 2). *Mycoplasma* was the most common bacterial genus found in the intestinal contents in all analyzed fish. This result is consistent with previous findings reporting the strong dominance of this genus in other salmonids including the Atlantic salmon (*Salmo salar*) (10). We detected TTGE bands corresponding to the same genera, but collected at different positions on the gel in some fish; this may be explained by differences in the GC content (%) of the region amplified (*i.e. Mycoplasma moatsii* showed three different bands with 46.4%, 51.5%, and 51.6% of GC, respectively, *versus* uncultured *Mycoplasma* showing only one band with 46.4% of GC). None of the dominant bacterial genera detected in the swim bladder resident microbiota were part of the trout distal gut microbiota. This result suggests a potential selection pressure imposed by the anatomic compartment, for example, via physicochemical characteristics, including oxygen tension (*i.e.* aerobiosis *versus* anaerobiosis), pH values (acid *versus* basic), and nutrient availability. These physicochemical characteristics create ecological niches for specific bacteria that will prevail under specific conditions. The differences observed in bacterial composition and diversity between the swim bladder and distal gut may be partially explained by differences in oxygen tension in each compartment. Anaerobiosis prevails in the gastro-enteric tract, implying that obligate and facultative anaerobes are more prominent under these conditions than those in the swim bladder. In contrast, the swim bladder mucosa is exposed to almost constant hyperoxia due to oxygen being present at high levels in the swim bladder chamber (15). High oxygen concentrations strongly promote the production of reactive oxygen species (superoxide and H_2_O_2_), which impairs microorganism growth by inducing oxidative damage in several cellular components, such as proteins and DNA (1). The swim bladder hyperoxic environment may impose a pro-oxidative challenge to some bacteria, favoring those capable of dealing with high oxygen tension (*i.e.* facultative and obligate aerobes), for example, by up-regulating antioxidant defensive mechanisms. These pro-oxidant conditions may function as a restrictive mechanism for bacterial growth in the swim bladder of physostomes. In this regard, both of the bacterial genera detected in the swim bladder resident microbiota exhibited the capacity to deal with oxygen. For example, species from *Arthrobacter* are obligate aerobes and *Cellulosimicrobium* species may be obligate or facultative anaerobes (6, 19). On the other hand, in physostomes, antioxidant defensive mechanisms, including the activity of antioxidant enzymes, such as catalase, glutathione peroxidase, and superoxide dismutase as well as the concentration of total glutathione (GSH+GSSG), have been reported to be stronger in the swim bladder than in other organs (16, 18). Species from *Arthrobacter* including *A. globiformis* may produce glutamic acid from sugars (16). Glutamic acid is one of the three amino acids required to synthesize glutathione, a major intracellular antioxidant molecule required for several cell survival signaling pathways (5, 21). Moreover, glutathione is a major intracellular antioxidant because it maintains tight control of the redox status in eukaryote cells and bacteria (7, 22). It currently remains unclear whether symbiosis exists between bacteria from *Arthrobacter* and physostomes. In this regard, the host swim bladder mucosa provides shelter and nutrients via mucus secretion and/or cellular detritus, which promotes bacteria survival, while bacteria may 1) compete with opportunistic pathogens for ecological niches or 2) provide glutamate or other molecules that may enforce the antioxidant defensive mechanism of the swim bladder against the pro-oxidative hyperoxic environment. Another factor potentially contributing to the differences observed between the swim bladder and distal gut is nutrient availability. Differences in nutrient availability and substrate affinity may contribute to competitive fitness, thereby affecting the microbial community structure (3). In this regard, nutrient availability is greater in the distal gut lumen than in the swim bladder mucosa, in which bacteria solely depend on cell mucosal secretions and cellular detritus.

The mechanisms by which bacteria access the epithelial mucosa of the swim bladder of physostomes have not yet been elucidated in detail. The structures of the respiratory system were previously considered to be sterile unless infected; however, a shift towards molecular methods for the quantification and sequencing of bacterial DNA revealed that these structures harbor a unique steady-state microbiota (11). Recent studies provided strong evidence to show that the lower respiratory tract harbors diverse communities of bacteria under healthy and diseased states, which suggests that a residual microbiota coexists in the host lung mucosa (4, 20).

Further studies are needed in order to investigate whether a potential swim bladder-gut axis exists via crosstalk of the swim bladder resident microbiota and gut microbiota, and the findings obtained will provide a better understanding of how microbes modulate fish health by regulating physiology, metabolism, and immunity. This appears to be of importance because evidence suggests the lung microbiota is involved in immune-mediated crosstalk with the gut microbiota as part of a “lung-gut axis” in mammalian species, which potentially influences the physiology of different organs, including the brain and liver (2).

## Figures and Tables

**Fig. 1:**
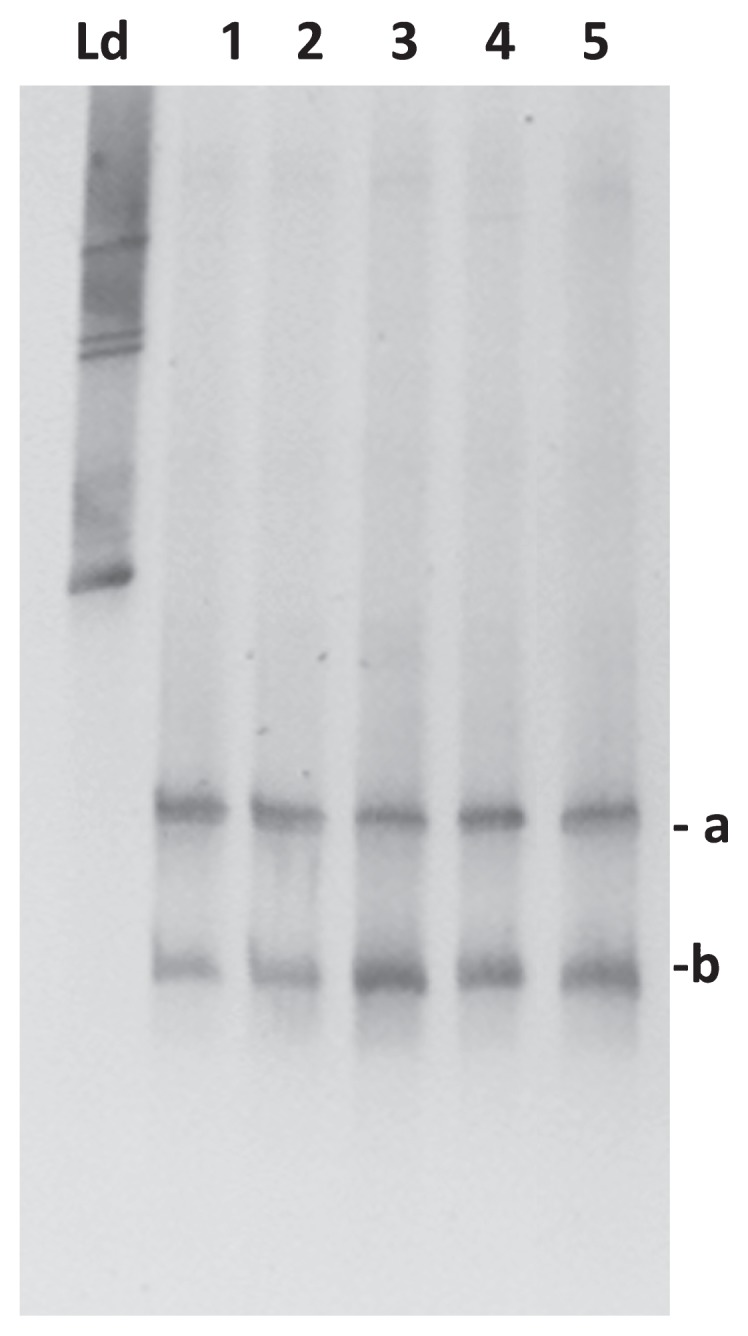
TTGE fingerprinting of the swim bladder microbiota of rainbow trout (*Oncorhynchus mykiss*). TTGE profiles based on the amplification of the V3–V4 region of 16S rRNA genes from DNA extraction from different individuals (*n*=5; Ld, Ladder). Bands a and b correspond to *Arthrobacter globiformis* and *Cellulosimicrobium* sp., respectively.

**Fig. 2:**
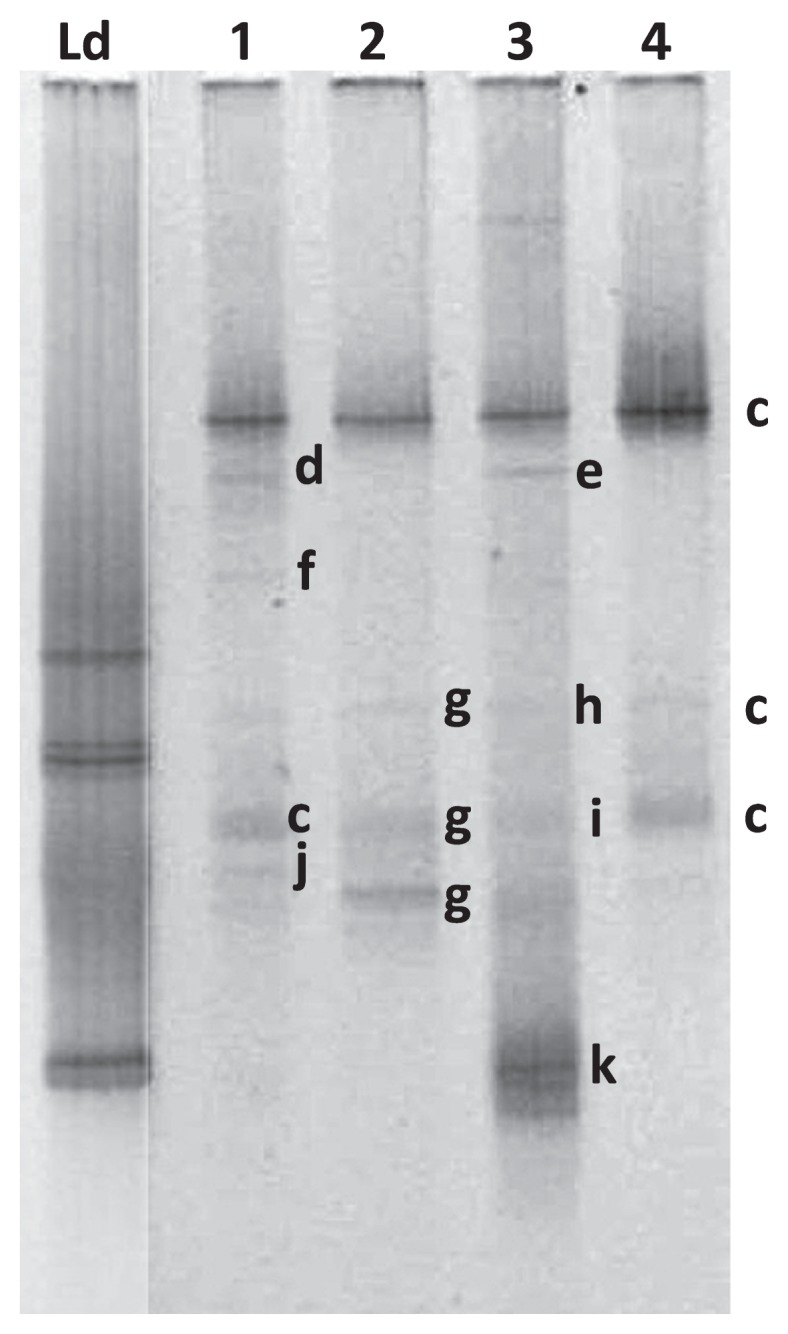
TTGE fingerprinting of intestinal contents of the microbiota of rainbow trout (*Oncorhynchus mykiss*). Comparisons of TTGE profiles based on the amplification of the V3–V4 region of 16 S rRNA genes from different individuals (*n*=4). Bands correspond to: Ld, Ladder; c, *Mycoplasma globiformis*; d, Uncultured *Brevinema* sp.; e, Uncultured *Cetobacterium* sp.; f, *Eubacterium* sp.; g, *Pseudomonas plecoglossicida*; h, *Aliivibrio wodanis*; i, *Vibrio ezurae*; j, Uncultured *Anaerofilum* sp.; k, Uncultured *Vibrio* sp.

**Table 1:** Nearest-match identification of TTGE bands (swim bladder and gut contents) to known sequences in the RDP II database

Band	Closest relative (Accession number)	Phylum	Class	x/y5)	Accession number6)
a	*Arthrobacter globiformis* (AB089841)1),2)	*Actinobacteria*	*Actinobacteria*	5/5	MF787227
b	*Cellulosimicrobium* sp. (AB188222)1),2)	*Actinobacteria*	*Actinobacteria*	5/5	MF787228
c	*Mycoplasma moatsii* (AF412984)3),4)	*Tenericutes*	*Mollicutes*	3/4	MF787230
c	Uncultured *Mycoplasma* sp. (FJ456770)3),4)	*Tenericutes*	*Mollicutes*	1/4	MF787229
d	Uncultured *Brevinema* sp. (HM630215)3),4)	*Spirochaetes*	*Spirochaetia*	1/4	MF787231
e	Uncultured *Cetobacterium* sp. (FM995174)3),4)	*Fusobacteria*	*Fusobacteriia*	1/4	MF787232
f	*Eubacterium* sp. (AJ629069)3),4)	*Firmicutes*	*Erysipelotrichia*	1/4	MF787233
g	*Pseudomonas plecoglossicida* (KC207084)3),4)	*Proteobacteria*	*Gammaproteobacteria*	1/4	MF787234
h	*Aliivibrio wodanis* (LN554846)3),4)	*Proteobacteria*	*Gammaproteobacteria*	1/4	MF787235
i	*Vibrio ezurae* (AY426981)3),4)	*Proteobacteria*	*Gammaproteobacteria*	1/4	MF787236
j	Uncultured *Anaerofilum* sp. (HG970996)3),4)	*Firmicutes*	*Clostridia*	1/4	MF787237
k	Uncultured *Vibrio* sp. (FM995173)3),4)	*Proteobacteria*	*Gammaproteobacteria*	1/4	MF787238

1) Found in the swim bladder.

2) >95% identity.

3) Found in intestinal contents.

4) >90% identity.

5) x, number of individuals with this microorganism; y, total number of individuals analyzed (*n*=5).

6) Sequence accession number deposited in GenBank.
